# Physiological Integration Ameliorates Negative Effects of Drought Stress in the Clonal Herb *Fragaria orientalis*

**DOI:** 10.1371/journal.pone.0044221

**Published:** 2012-09-05

**Authors:** Yunchun Zhang, Qiaoying Zhang, Marek Sammul

**Affiliations:** 1 Department of Botany, Institute of Agricultural and Environmental Sciences, Estonian University of Life Sciences, Tartu, Estonia; 2 Shandong Polytechnic University, Jinan, Shandong Province, P. R. China; 3 Department of Botany, Institute of Ecology and Earth Sciences, University of Tartu, Tartu, Estonia; Brigham Young University, United States of America

## Abstract

Clonal growth allows plants to spread horizontally and to establish ramets in sites of contrasting resource status. If ramets remain physiologically integrated, clones in heterogeneous environments can act as cooperative systems – effects of stress on one ramet can be ameliorated by another connected ramet inhabiting benign conditions. But little is known about the effects of patch contrast on physiological integration of clonal plants and no study has addressed its effects on physiological traits like osmolytes, reactive oxygen intermediates and antioxidant enzymes. We examined the effect of physiological integration on survival, growth and stress indicators such as osmolytes, reactive oxygen intermediates (ROIs) and antioxidant enzymes in a clonal plant, *Fragaria orientalis*, growing in homogenous and heterogeneous environments differing in patch contrast of water availability (1 homogeneous (no contrast) group; 2 low contrast group; 3 high contrast group). Drought stress markedly reduced the survival and growth of the severed ramets of *F. orientalis*, especially in high contrast treatments. Support from a ramet growing in benign patch considerably reduced drought stress and enhanced growth of ramets in dry patches. The larger the contrast between water availability, the larger the amount of support the depending ramet received from the supporting one. This support strongly affected the growth of the supporting ramet, but not to an extent to cause increase in stress indicators. We also found indication of costs related to maintenance of physiological connection between ramets. Thus, the net benefit of physiological integration depends on the environment and integration between ramets of *F. orientalis* could be advantageous only in heterogeneous conditions with a high contrast.

## Introduction

One of the features of clonal growth in plants is the capacity to exchange resources, such as water, photo-assimilates and nutrients [1]–[8] and non-resource agents, such as defence compounds, signalling molecules or pathogen agents [9]–[11] between interconnected ramets. Such physiological integration between potentially independent ramets is more important when different ramets within one clonal fragment experience heterogeneity of microhabitats [4], [12], [13]. Exchange of resources within a clone, i.e. support to one ramet from another one, may buffer the negative effects of individual microhabitats [3], [7], [13]–[16]. This buffer effect may also be accompanied with an improvement of physiological traits like osmolytes, reactive oxygen intermediates and antioxidant enzymes, but these effects have been little studied in clonal plants. Physiological integration may also have disadvantages such as energy cost of maintenance of inter-ramet connections and rapid spread of pathogens throughout the system of interconnected ramets [14], [17]–[20]. In fact, some studies have reported that under chronic or severe stress integration may result in lower fitness, and clonal plants may cease to support dependent ramets [21]–[24], whereas others have found that clones continue to support dependent ramets despite prolonged stress [3], [4], [15], [25], [26]. Intraclonal resource translocation may be mainly driven by the degree of difference (contrast, [4], [27]) between adjacent patches [28], [29], except in cases of developmental constraints (e.g., support to establishing ramets by an adult part of a clone [30], [31]. However, experimental evidence for this positive relationship between contrast and the strength of clonal integration is rather scarce [3], [4], [15], despite the fact that the level of contrast probably determines the balance between costs and benefits of physiological integration.

Water deficit is a major constraint for plant survival. In natural habitats, water often has a patchy distribution on the scale relevant to individual plants, so that neighbouring ramets of clonal plants may experience contrasting levels of water availability [32]–[34]. In clonal plants, physiological integration allows water to be transported from connected and well-watered ramets to ramets subjected to drought, thereby alleviating water stress in water-stressed ramets under heterogeneous water environments [15], [35]–[37]. Several studies have shown the effects of variable water availability on the morphology and gas exchange of clonal plants [3], [15], [36], [38]–[43]. However, no study has separated the effect of water stress on clonal plant from the effect of provision of support to a dependent ramet by a supporting one.

Osmolytes (osmoregulation substances) and reactive oxygen intermediates (ROIs) have been used as signals of drought stress in many studies of non-clonal plants [44]–[48]. Moreover, the level of stress can also be evaluated by estimation of the activity of antioxidant enzymes, which can clean out excessive active oxygen resulting from stress [47], [49]–[51]. Stress induced damage to plant cells can also be evaluated by measuring free radical induced peroxidation of lipid membranes [52]. The malondialdehyde (MDA) content is an indicator of membrane lipid peroxidation. This damage to membranes is also associated with accumulation of H_2_O_2_ [48], [53], [54]. Free proline content may serve as a means of osmotic adjustment, it could function as a hydroxyl radical scavenger to prevent membrane damage and protein de-naturation [55]. Plants also have evolved antioxidative enzymes, including peroxidase (POD), superoxide dismutase (SOD), ascorbate peroxidase (APX) and catalase (CAT), to protect plants against oxidative damage [47]. Above-mentioned stress-indicator traits have been extensively documented in non-clonal plants, but so far little study has been done in clonal plants.

In this study, we examine the effect of physiological integration between ramets and the level of contrast in water availability between patches on survival, growth, and level of stress on a clonal plant *Fragaria orientalis*. We specifically address the following questions: (1) Are the survival, growth, and level of stress of supporting ramets growing in well-watered soils affected by integration with dependent ramets growing in water shortage? I.e., how large is the cost of integration for a supporting ramet? (2) Is the level of support to a dependent ramet affected by the water availability contrast between patches inhabited by supporting and dependent ramet? We expect that the physiological integration ameliorates the negative effect of drought stress on dependent ramets, while the net benefit of integration on the whole clonal fragment increases with increasing contrast between patches inhabited by supporting and dependent ramets.

## Materials and Methods

### Plants and Experimental Design

*Fragaria orientalis* (Rosaceae) is a stoloniferous, perennial, rosette herb which is widely distributed in Korea, Mongolia, Eastern Russia and China. In China, it is common in North China and Eastern Qinghai-Tibetan Plateau, inhabiting forests and meadows on mountain slopes [56], [57]. The axillary buds on the vertical stems may grow out and form stolons. The stolons usually take root on stem nodes when reaching a moist substratum, and even single stem node can establish and grow as a ramet.

**Table 1:** Experimental design.

Treatment	Supporting ramet	Stolon connection	Dependent ramet
Homogeneity (Ho)	90% FC	Intact	90% FC
	90% FC	Severed	90% FC
Low contrast (L)	90% FC	Intact	60% FC
	90% FC	Severed	60% FC
High contrast (H)	90% FC	Intact	30% FC
	90% FC	Severed	30% FC

At the start of the experiment, 15 plants of *F. orientalis*, each consisting of more than 12 newly produced ramets (on stolons), were excavated around Maoxian Ecological Station, Chinese Academy of Sciences (31°41′07″N, 103°53′58″E; 1,816 m asl.). The sampling site did not belong to the part of any farms or national parks. *Fragaria orientalis* is widespread in China and it is not an endangered or protected species, so we did not need any relevant permissions/permits for plant samples collection. The plants were collected at least 1000 m away from one another, and were thus considered as 15 distinct genotypes [58]. These original plants were dissected into clonal fragments, each fragment composed of two interconnected ramets of similar size. One ramet in each pair was referred to as the initial proximal part (called the “supporting ramet”; it is well-watered and intended to provide water to the distal ramet in the experiment), indicating its relative proximity to the mother rosette, while the other as the initial distal part (called the “dependent ramet”; it is intended to receive water from the supporting ramet in the experiment). With the stolon still intact between two ramets, these clonal fragments were planted in trays of sand for 20 days. Once well established (rooted), they were size-standardized by removing extra leaves so that only three youngest leaves remained, and transplanted into plastic pots (20 cm in diameter and 15 cm in height) filled with homogenized soil to a depth of 14 cm. Leaf removal could affect the source-sink relationship of individual ramets (e.g., the balance between above and belowground growth), but it would keep the source-sink relationship between the two interconnected ramets before the start of the experiment the same across all the treatments, which is crucial for the study of the effects of physiological integration [15], [58]–[60]. The supporting and dependent ramets of each clonal fragment were planted in separate pots (forming a pair of pots), and they were connected by an undamaged stolon. In half of the pots the stolon was cut off (severed treatment). Intact connection between paired ramets (intact stolon) allowed physiological integration between ramets, while in the severed treatments physiological integration was impeded. Plants were grown in a glasshouse under a semi-controlled environment, with the day temperature range of 12–31°C, night temperature range of 9–15°C, and the relative humidity range of 35–85%. After one week, all ramets were size-standardized again by removing all leaves except the youngest one so that foliage of similar area remained.

**Figure 1:**
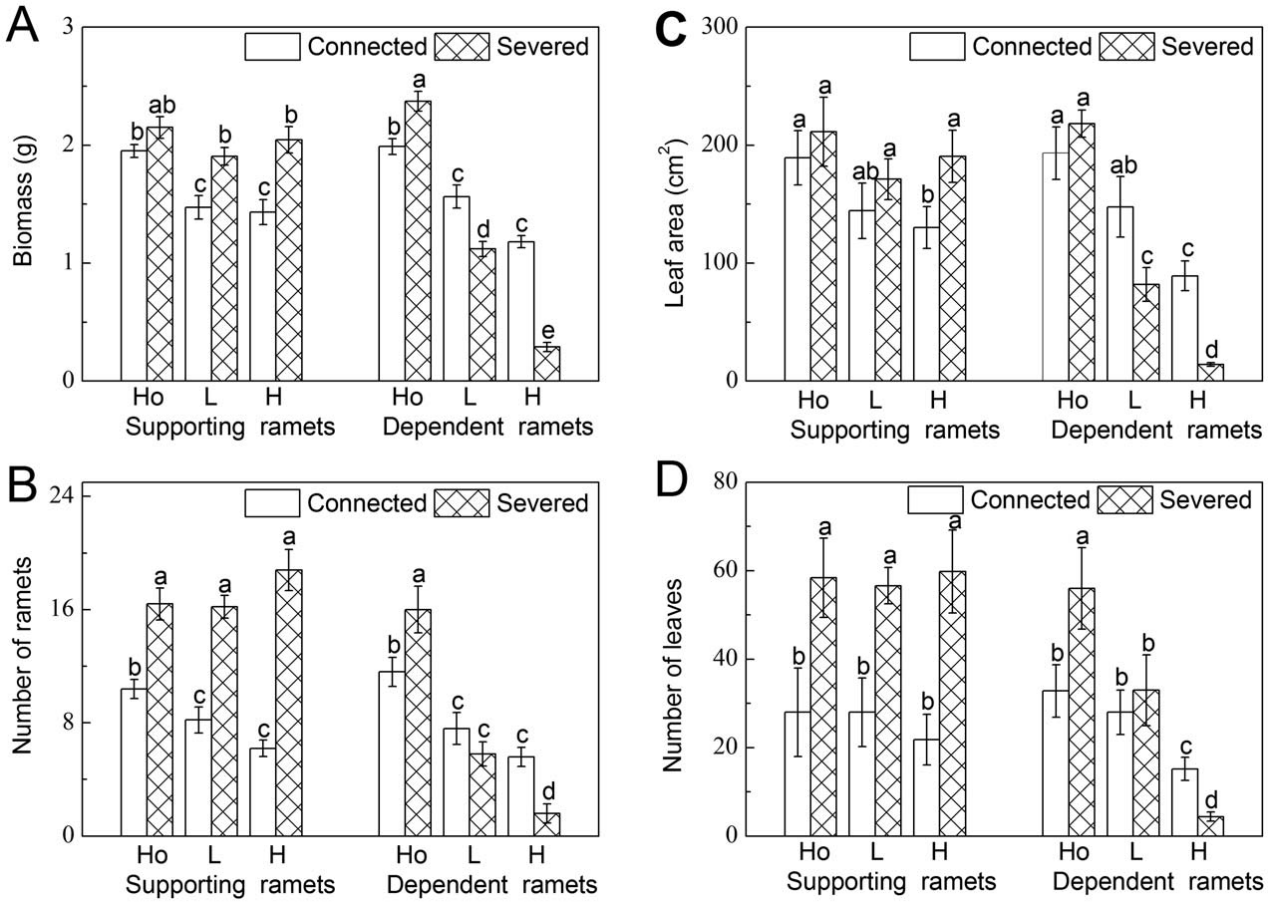
Growth parameters. (A) Biomass, (B) number of ramets, (C) leaf area, and (D) number of leaves of the supporting and the dependent ramets. Data are means±SE (n = 15). Bars sharing the same lowercase letter are not different at p = 0.05. Treatments are coded as in Table 1.

**Table 2:** F-values of two-way ANOVA which was used to test for the effects of contrast level of water availability (C), severance of stolon connection between the ramets (S) and their interaction (C*S) on the growth parameters and levels of stress indicators in supporting and dependent ramets.

Characters	Supporting ramets			Dependent ramets		
	C	S	C*S	C	S	C*S
Biomass	16.045^**^	54.316^**^	4.567^*^	572.274^**^	105.448^**^	127.356^**^
No. of ramets	1.511^ns^	203.106^**^	8.487^**^	169.511^**^	0.849^ns^	29.545^**^
Leaf area	6.682^**^	12.035^**^	1.281^ns^	119.755^**^	23.016^**^	16.985^**^
No. of leaves	4.196^*^	63.412^**^	7.467^**^	43.832^**^	4.037^*^	10.924^**^
Proline	0.299^ns^	0.054^ns^	2.072^ns^	276.829^**^	557.667^**^	231.767^**^
MDA	2.538^ns^	0.938^ns^	1.436^ns^	80.027^**^	179.396^**^	67.987^**^
H_2_O_2_	0.388^ns^	0.275^ns^	1.321^ns^	50.712^**^	32.914^**^	12.723^**^
Protein	0.600^ns^	0.891^ns^	0.679^ns^	17.631^**^	20.605^**^	15.253^**^
POD	0.031^ns^	1.039^ns^	2.422^ns^	87.597^**^	212.281^**^	56.125^**^
SOD	0.989^ns^	1.717^ns^	2.347^ns^	178.863^**^	219.330^**^	106.120^**^
APX	1.577^ns^	2.211^ns^	3.024^ns^	122.231^**^	89.227^**^	84.107^**^
CAT	1.099^ns^	0.370^ns^	1.332^ns^	353.226^**^	815.577^**^	308.031^**^

For the supporting part, df (degrees of freedom) C, df S and df C*S are (2, 90), (1, 90), (2, 90), respectively. Because ramets of the severed dependent part died in high contrast, this treatment was not included in the ANOVA analysis for the dependent part. So df C, df S and df C*S are (2, 82), (1, 82), (2, 82), respectively. Significance level: ns P>0.05, *P<0.05, **P<0.01.

At the beginning of the experiment, all ramets were about 2 cm tall. Both the unsevered and severed pairs of ramets were divided into three groups: 1) homogeneous group, both ramets of each pair were well-watered [up to 90% of field capacity (FC)]; 2) low contrast group, the supporting ramet of each pair was well-watered [90% of FC] and the dependent ramet was subject to less-watered treatment [60% of FC]; 3) high contrast group, the supporting ramet was well-watered [90% of FC] and the dependent ramet was subject to severe drought [30% of FC] (Table 1). Thus, six different treatments were formed (two levels of physiological integration × three levels of water availability contrast). In each treatment, there were 15 replicates, each of which was derived from one of the 15 original rosettes.

**Figure 2:**
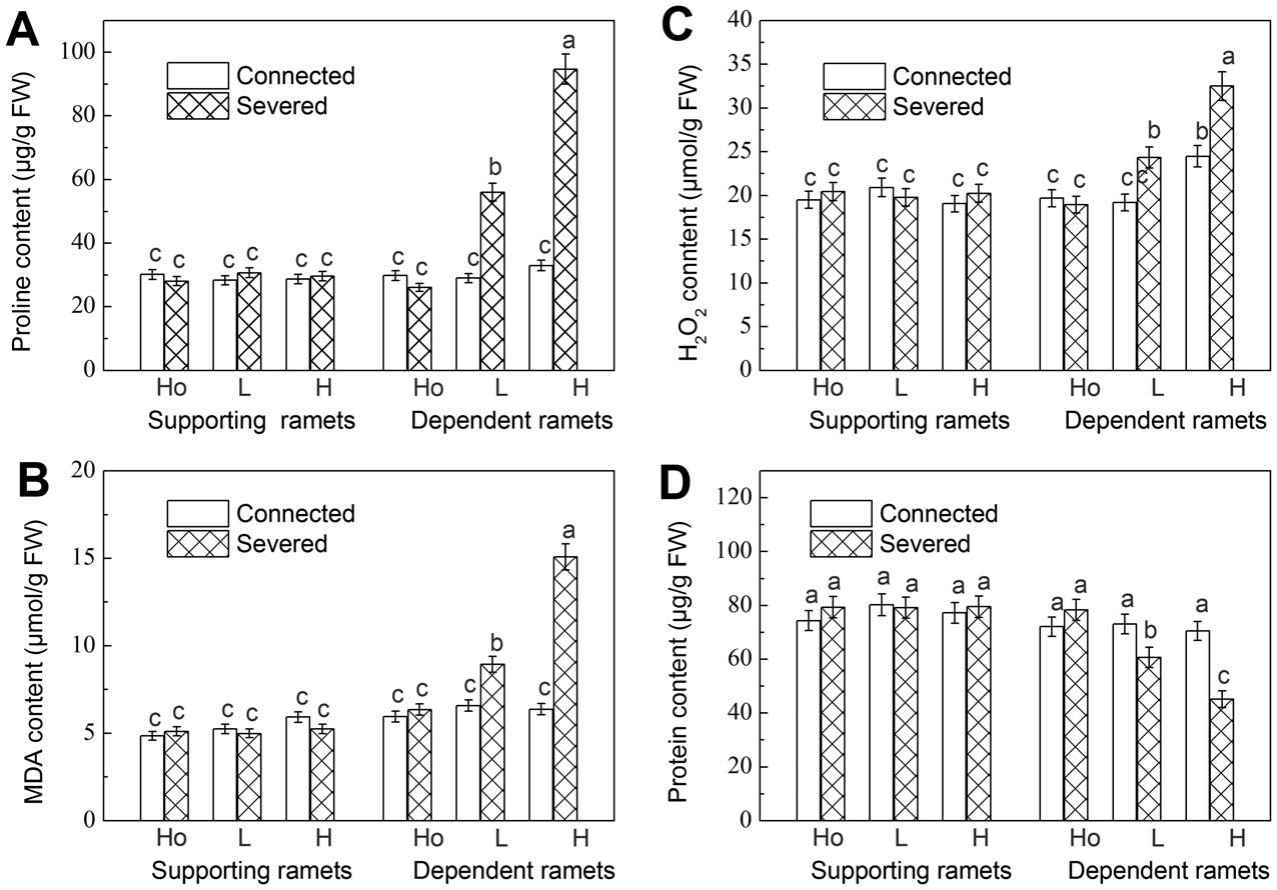
Osmolytes and ROIs. (A) Proline content, (B) MDA content, (C) H_2_O_2_ content and (D) protein content of the supporting and the dependent ramets. Data are means±SE (n = 15). Bars sharing the same lowercase letter are not different at p = 0.05. Treatments are coded as in Table 1.

In each treatment, the pots were re-watered to their respective FC by replacing the amount of water transpired every second day. Evaporation from the soil surface was reduced by enclosing all pots in plastic bags sealed at the base of the stem of each ramet. The amount of water was determined by weighing the pots. An empirical relationship between plant fresh weight (g) and plant leaf area (cm^2^) was used to correct pot water for changes in plant biomass. In addition, 15 additional control pots were planted with dead *F. orientalis* and the pots enclosed in plastic bags in the same way as other treatments. These pots were also weighted every second day in order to estimate evaporation from the soil surface. A total 8 g of slow-release fertilizer (13% N, 10% P and 14% K) was added to each pot during the experiment. After 100 days, samples of the youngest fully expanded leaves were taken from the original ramets of each pot for determination of physiological indicator (osmolytes, ROIs and antioxidant enzyme activity). At the end of the experiment (the experiment lasted 110 days), all parts of each plant in each pot were marked and harvested.

**Figure 3:**
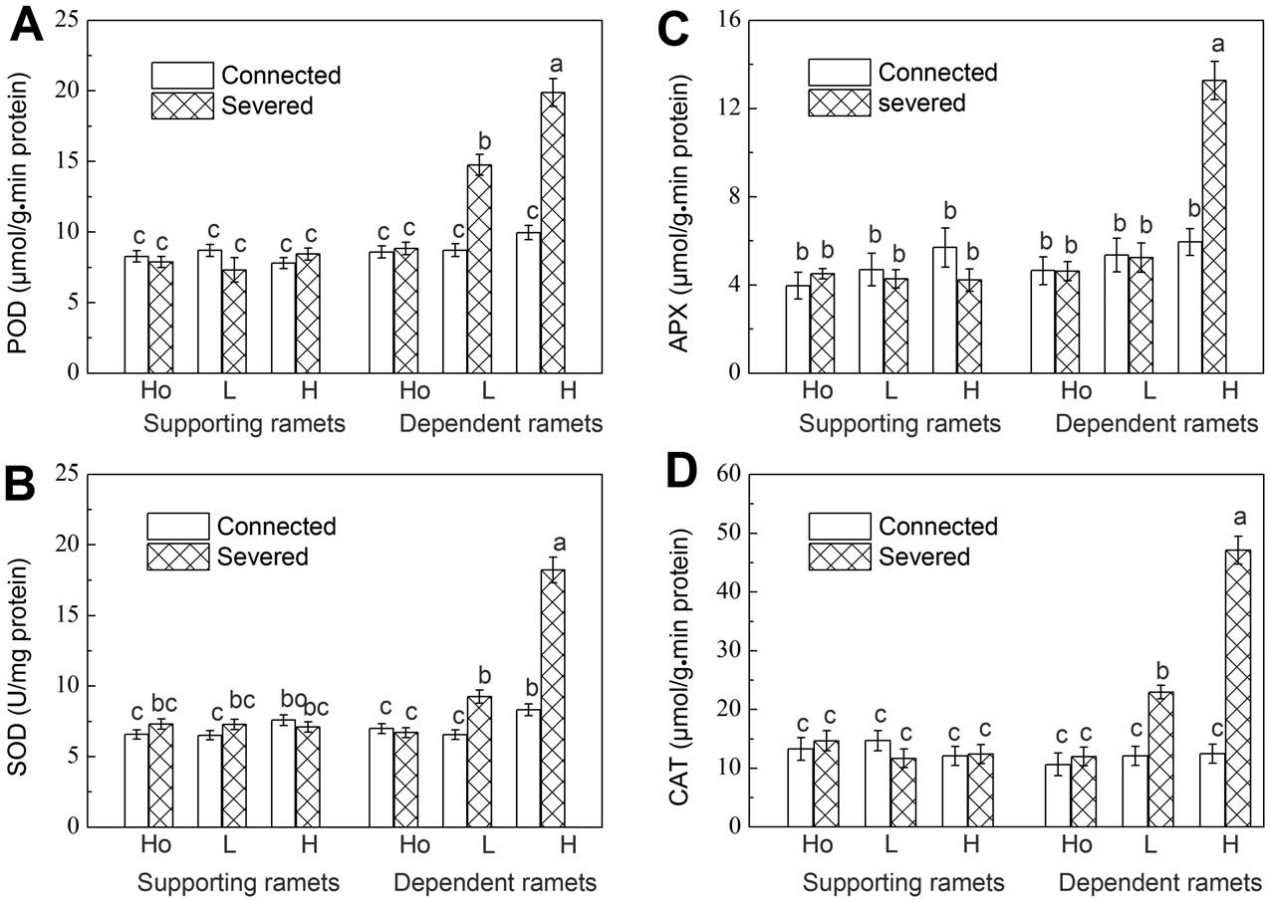
Antioxidant enzymes activity. (A) POD, (B) SOD, (C) APX and (D) CAT of the supporting and the dependent ramets. Data are means±SE (n = 15). Bars sharing the same lowercase letter are not different at p = 0.05. Treatments are coded as in Table 1.

### Survival and Growth

At the end of the experiment, mortality rates were determined in supporting and dependent ramets, total number of new ramets produced by original ramet and leaves per pot were counted, total leaf area per pot was measured using a CI-203 Laser Area Meter (CID Inc.), and the biomass per pot was determined after drying the plants at 70°C for 48 h.

### Osmolytes and ROIs

Osmolytes and ROIs were estimated by the contents of proline, malondialdehyde (MDA), H_2_O_2_ and total soluble protein.

The free proline content was determined according to the method described by Bates *et al*. [61]. The absorbance of the free proline concentration was measured at 520 nm. The MDA content was determined by the thiobarbituric acid (TBA) reaction as described by Heath and Packer [62]. The absorbance of MDA was measured at 532, 600 and 450 nm. The MDA content was calculated according to the formula: MDA [µM]  = 6.45(A_532_–A_600_)–0.56A_450_. The H_2_O_2_ content was determined according to Prochazkova *et al*. [63]. The absorbance of the H_2_O_2_ concentration was measured at 415 nm. Total soluble protein content was determined using Coomassie brilliant blue followed the Bradford assay method [64]. Preparation of crude extract was based on the method of Pinto *et al*. [65].

### Antioxidant Enzymes Activity

Antioxidant enzyme activities of ramets were estimated from the activities of peroxidase (POD), superoxide dismutase (SOD), ascorbate peroxidase (APX) and catalase (CAT).

Extracts for the determination of antioxidant enzyme activities were prepared from 1.0 g of fully developed new leaves homogenized under ice-cold conditions in 3 ml of extraction buffer, containing 50 mM phosphate buffer (pH 7.4), 1 mM EDTA, 1 g PVP and 0.5% (v/v) Triton X-100. The homogenates were centrifuged at 10,000**×**g for 30 min and the supernatant was used for the assays.

POD activity (EC 1.11.1.7) was based on the determination of guaiacol oxidation, as described by Ekmekci and Terzioglu [66]. Activity was determined by the increase in absorbance at 470 nm due to guaiacol oxidation. SOD activity (EC 1.15.1.1) was assayed by the inhibition of the photochemical reduction of nitroblue tetrazolium (NBT) [67]. The reduction in NBT was followed by reading absorbance at 560 nm. One unit of SOD was defined as the amount of enzyme inhibiting the photo-reduction of NBT by 50% [68]. APX activity (EC 1.11.1.11) was assayed followed Nakano and Asada [69]. The hydrogen peroxide-dependent oxidation of ascorbate was followed by decrease in the absorbance decrease at 290 nm. CAT activity (EC 1.11.1.6) was determined in the homogenates by measuring the decrease in absorption at 240 nm [70]. The CAT activity was calculated using the extinction coefficient for H_2_O_2_.

### Statistical Analysis

We used two-way ANOVA to assess the effects of water availability contrast (three levels) and severance of stolon connection (severed and intact) on all variables of supporting and dependent ramets. The variables include biomass, number of ramets, leaf area, number of leaves, proline content, MDA content, H_2_O_2_ content, protein content, POD, SOD, APX and CAT. In the two-way ANOVA models, water availability contrast and severance of stolon connection were treated as fixed factors. The data met the model assumptions of normality and homoscedasticity and thus were not transformed before analysis. The Duncan test was used to compare the means among the treatments. All statistical analyses were done with the SPSS 15 for Windows statistical software package.

## Results

### Survival and Growth

At the end of the experiment, 53% (8 ramets of 15 ramets) of severed dependent ramets in high water contrast died. All other ramets survived.

Supporting ramets with an intact stolon had, when compared to severed ramets, lower biomass, fewer ramets and leaves in heterogeneous environments (low contrast and high contrast) and smaller leaf area in high contrast treatments (Table 2 and Fig. 1A, B, C, D). There was no effect of severing on biomass of supporting ramets in the homogeneous treatments, and on leaf area in the homogeneous and low contrast treatments (Fig. 1A, C). Compared to intact ramets, severed dependent ramets had higher biomass, more ramets and more leaves in the homogeneous treatments but lower biomass and leaf area in the heterogeneous ones. Severed dependent ramets also had fewer ramets and leaves than intact dependent ramets in high contrast (Table 2 and Fig. 1A, B, C, D). There were no significant differences in leaf area in the homogeneous treatments as well as number of ramets and number of leaves in low contrast (Fig. 1B, C, D).

Biomass, number of ramets and leaf area of connected supporting ramets in the heterogeneous treatments were smaller than those in the homogeneous ones. There was no effect of water availability contrast on any growth parameter of the severed ramets (Table 2 and Fig. 1A, B, C, D). Biomass, number of ramets, leaf area and number of leaves of both connected and severed dependent ramets decreased with the increasing contrast, while the degree of the decrease was higher in severed ramets than in connected ramets (Table 2 and Fig. 1A, B, C, D).

### Osmolytes and ROIs

Proline, MDA, H_2_O_2_ and soluble protein content of both connected and severed supporting ramets were not significantly different between the three water availability contrasts. There were also no significant differences in these parameters between connected and severed supporting ramets. In dependent ramets, proline, MDA and H_2_O_2_ of severed ramets increased and soluble protein content decreased with the increase of the contrast level. Proline, MDA and H_2_O_2_ content of severed ramets was significantly higher, while soluble protein content was significantly lower than in connected ramets in low and high contrast, and there was no difference in the homogeneous treatments (Table 2 and Fig. 2).

### Antioxidant Enzymes Activity

There were no significant differences between connected and severed ramets or among the contrasts in the levels of POD, SOD, APX and CAT of the supporting ramets. For dependent ramets, POD, SOD and CAT content in severed ramets was higher than in connected ramets in the heterogeneous treatments and APX content in high contrast. POD, SOD, APX and CAT levels in the homogeneous treatments and APX content in low contrast showed no significant differences between severing and non-severing treatments. There were no significant differences between different contrast levels in POD, APX and CAT content of connected ramets. But the SOD content of connected ramets in high contrast was higher than that in low contrast and homogeneous treatments. POD, SOD and CAT content in severed ramets increased with increasing contrast level. APX content in severed dependent ramets was higher than in connected dependent ramets in high contrast, but there was no difference in the homogeneous and low contrast treatments (Table 2 and Fig. 3).

## Discussion

### Drought Induced Stress

Our study clearly indicates an increased stress in plants with increasing shortage of water and this response is very consistent regardless of the size characteristic or physiological indicator studied. Severed plants subjected to stress without the support from a ramet grown in benign conditions were smaller and had fewer leaves as well as less clonal offspring ramets (Fig. 1). The effect was proportional to the level of induced stress.

To cope with an increased water shortage, plants may adjust their osmotic potential. Free proline content may serve as a means of osmotic adjustment, and improve water relations under drought conditions [71]. In addition, it could function as a hydroxyl radical scavenger to prevent membrane damage and protein denaturation [55]. Higher content of free proline has been reported in many drought-induced plants [71]–[73]. We found a greater proline accumulation in severed dependent ramets subjected to water shortage (Fig. 2A). Thus, plants may adjust to water shortage by increasing proline content, but reduced protein content in dry conditions (Fig. 2D) indicates that they could only adjust to a certain extent and damage could not be avoided.

In our study, both MDA and H_2_O_2_ increased with increasing water shortage in severed ramets, which indicates that plants sustained considerable damage and that damage was proportional to water shortage. Their increase during drought stress has been reported in non-clonal plants as indicators of drought stress [48], [71], [74]. We also found a clear increase in POD, SOD, APX, and CAT levels in stressful conditions, which is consistent with the findings in many non-clonal plants during drought stress [47], [75], [76]. POD, SOD, APX and CAT are important enzymes which protect plants against oxidative damage. Their increase in our study can be interpreted as a measure of effort plants put into repair. This repair will be costly to plants and is not entirely successful. Our results showed that when water availability is reduced to as low as 30% of soil water field capacity this has markedly negative effects even on the survival of *F. orientalis*. During the experiment 53% of severed dependent ramets growing in such conditions died, indicating that drought was a major stress factor in the model environment when ramets where denied support from a physiologically integrated ramet growing in benign conditions.

### Benefits from Physiological Integration

Ramets of *F. orientalis* in favourable conditions provide support to connected drought stressed ramets. This result is consistent with several studies with other clonal species [37], [39], [40], [77]–[84] where it was found that drought had negative effects on severed ramets, and the negative effects were ameliorated in connected ramets. In our case with *F. orientalis* the support by a supporting ramet was so efficient that the level of most of the stress hormones did not increase in the dependent ramet. Only the levels of H_2_O_2_, protein content, and SOD in the high-contrast treatments increased in connected dependent ramets. However, the growth of connected ramets was reduced even in the low-contrast treatments, showing that water shortage affected them, even though stress was not detected in the chemical analyses of these plants.

In the case of extremely severe drought or in conditions of chronic water shortage clonal plants may cease to support dependent ramets [21], [23], [24]. We can conclude that in our experiment the support to dependent ramets of *F. orientalis* never stopped. The higher the contrast between paired patches, the stronger was the support to a dependent ramet (as indicated by the increasing difference between severed and connected ramets). Thus, water sharing between connected ramets was very efficient [25], [38], [77], [78], [83] and it increased with increasing demand.

### Cost of Support

Apart from benefits, integration may also have a cost for ramets exporting resources, especially when the supported ramets are located in more stressful conditions [85]. In our study, supporting ramets severed from a dependent ramet were always bigger than supporting ramets with intact stolon connection (Fig. 1), and this difference is most obvious in the number of leaves and ramets. To a lesser extent this also holds true for severed dependent ramets. The fact that connected ramets were smaller even when both of them received the same amount of water indicates the metabolic costs of maintaining the connection between ramets [15], [19], [85]. This cost was also shared between both proximal and distal ramets even though the former seemed to bear the greater share. The cost was surprisingly large, if the increase in number of modules (ramets or leaves) in severed ramets was an indication of maintenance costs of a connection. However, the increase in number of modules in severed ramets may be also an indication of a release from apical dominance.

When clonal fragments of *F. orientalis* were partially exposed to drought, and stolon connections between the droughty and wet ramets remained intact, clonal integration showed great benefits to the ramets in the dry patches (which imported resources) and significant costs to the connected wet ramets (which exported resources). There was a clear increase in growth reduction in connected supporting ramets with increasing water deficiency in dependent ramets, but there were no differences in stress indicators between connected and severed supporting ramets. This indicates that translocation of substances to resource-deficient ramets clearly reduces performance of the supplying part of a clonal fragment [15], [19], [28], [85]. The effect is primarily associated with growth reduction, but supporting ramets do not export water at a level which would cause the increase in production of stress-related chemicals. Thus, we conclude that the support is not given at a level that would directly harm the supporting ramet. A possible explanation for this might be that vascular translocation of water may require expenditure of energy [86] and therefore is expected to affect the growth of supporting ramets. Removal of stem and shoot apex usually results in the growth of one or more of the lateral buds and production of more new shoots [87]. Thus another possible explanation is that the loss of apical dominance caused by stolon severance promotes the growth of supporting ramets. Significant costs of water export in clonal plants also have previously been reported in *C. flacca* and *Ficus tikoua* spanning a gradient in water availability [39], [43]. However, this contrasts with the results in *H. bonariensis* [37], *C. hirta* [39], *P. anserina* [80] and *Buchloe dactyloides* [88], which showed that clonal integration confers benefits on drought stressed ramets but no costs on the connected supporting ramets. The support from well-watered ramets to drought ramets is not given at a level that would directly harm the supporting ramets, even though we did not know whether the growth difference of supporting ramets connected to and severed from dependent ramets were caused by maintenance costs of a connection or a release from apical dominance.

### Conclusions

Our results, together with previous studies on the effect of clonal integration on morphology and photosynthesis of *F. orientalis* [3], [15], suggest that clonal integration can help *F. orientalis* to deal with drought stress, especially in conditions where contrast between patches is large. In such conditions the benefits from connection could outweigh the considerable costs of maintaining the clonal connection between ramets which make persistence of connections in homogeneous conditions disadvantageous. Clonal integration can therefore be understood as part of a stress tolerance strategy that enhances the survival and growth of clonal plants growing in patchy environments [4], [19], [89]–[91]. However, excessive integration can be adaptive only when its benefits outweigh the costs and not in every environment.
